# Antioxidant Content, Antioxidant Activity, and Antibacterial Activity of Five Plants from the Commelinaceae Family

**DOI:** 10.3390/antiox3040758

**Published:** 2014-11-17

**Authors:** Joash Ban Lee Tan, Wei Jin Yap, Shen Yeng Tan, Yau Yan Lim, Sui Mae Lee

**Affiliations:** School of Science, Monash University Sunway Campus, Bandar Sunway 46150, Malaysia; E-Mails: wjyap4@student.monash.edu (W.J.Y.); sytan89@student.monash.edu (S.Y.T.); lim.yau.yan@monash.edu (Y.Y.L.); lee.sui.mae@monash.edu (S.M.L.)

**Keywords:** Commelinaceae, antioxidant, antibacterial, methanolic extracts

## Abstract

Commelinaceae is a family of herbaceous flowering plants with many species used in ethnobotany, particularly in South America. However, thus far reports of their bioactivity are few and far between. The primary aim of this study was to quantify the antioxidant and antibacterial activity of five Commelinaceae methanolic leaf extracts. The antioxidant content was evaluated by the total phenolic content (TPC), total tannin content (TTC), and total flavonoid content (TFC) assays. The antioxidant activities measured were DPPH free radical scavenging (FRS), ferric reducing power (FRP), and ferrous ion chelating (FIC); of the five plants, the methanolic leaf extract of *Tradescantia zebrina* showed the highest antioxidant content and activity, and exhibited antibacterial activity against six species of Gram-positive and two species of Gram-negative bacteria in a range of 5–10 mg/mL based on the broth microdilution method.

## 1. Introduction

The Commelinaceae family comprises 37 genera and over 600 species [1] of monocotyledonous herbaceous flowering plants [2,3], notably the spiderworts (*Tradescantia* sp.). They are believed to have originated from the old world tropics, but now they are widely distributed throughout the sub-tropics and tropics of both hemispheres, with some species surviving even in more temperate climates [4]. These plants are often grown for ornamental purposes due to their bluish or purplish leaves and/or flowers, but are also known to be used ethnobotanically to treat many diseases, including mycosal infections [5], venereal diseases [6], wounds [7], gastrointestinal disorders, and cancer [8], which may be linked to their antibacterial and antioxidant properties. However, reports on the antibacterial and antioxidant properties of these plants have remained rare so far.

Plants of this family are a good source of renewable bioactive compounds, as members of the Commelinaceae are generally evergreen, perennial, hardy, and prolific. Like other silicon-accumulating plant families such as the Gramineae (rice/wheat) and Cucurbitales (pumpkin/squash), Commelinaceae are less prone to growth, development, and reproduction abnormalities than plants with less efficient silicon accumulation [9]. In fact, many species of the Commelinaceae are considered weeds and pests due to their rapid growth, hardiness, and ability to root at the nodes. They are also resilient to most herbicides, and able to rapidly regenerate if left untreated [4]. However, these same traits also make them an ideal renewable and abundant resource for bioactive compounds.

The antioxidant and antibacterial activity of *Rhoeo spathacea* (Swartz) Stearn leaves had been previously reported by our group [10]. In the present paper, two other cultivars of *R. spathacea* are reported; this represents the first time the bioactivity of these cultivars has been studied. The first is *Rhoeo bermudensis*, a dwarf cultivar of *R. spathacea* that is also known as *Tradescantia spathacea* “Hawaiian Dwarf”, or *Rhoeo spathacea nana.* It is physically similar to *R. spathacea* (Swartz) Stearn, albeit much smaller—its leaves are a mere 10–20 cm long, compared to 15–30 cm for *R. spathacea* (Swartz) Stearn. The overall plant grows to 30–45 cm tall, approximately 60% of the height of *R. spathacea* (Swartz) Stearn. Unlike *R. spathacea*, it lacks flowers, but is able to root readily at the nodes and spread across the ground rapidly, like many other species of the Commelinaceae family [4]. It is colloquially known as the Hawaiian dwarf, or dwarf oyster plant [11]. The second cultivar, *Rhoeo spathacea* var. variegata, is also known as *Rhoeo spathacea* vittata and bears resemblance to *R. spathacea* (Swartz) Stearn, but with yellow striations on the upper side of the leaves instead of the stark green upper side of the original cultivar [12].

*Tradescantia pallida* (Rose) D. R. Hunt., also known as purple heart or wandering jew, is a low-growing tetraploid plant that is shade-tolerant, able to thrive on various soil conditions, and possesses strong resistance to insects and parasites. These traits allow it to rapidly colonize various environments, acting as an invasive weed [13,14]. Therefore, it is well adapted and widely distributed in tropical and subtropical regions [15,16] and is commonly being grown as an ornamental, ground-covering, or hanging plant. Although also renowned for its ability to effectively remove volatile organic pollutants from the air [17], *T. pallida* has also been traditionally used as an anti-inflammatory and anti-toxic supplement, and to improve blood circulation [18].

Similarly, *Tradescantia zebrina* Bosse var. *zebrina*, a close relative to *T. pallida*, is similarly categorized as an invasive plant species, and commonly distributed in tropical and subtropical regions [19]. It has oval, pointed leaves, variegated green and silver stripes on the upper surface, with a purple underside [20,21]. *T. zebrina* is traditionally used to treat gastrointestinal disorders [22].Other studies have also reported that *T. zebrina* exhibits insecticidal properties [23] and can inhibit the 15-lipoxygenase pathway involved in asthmatic attacks [24].

*Callisia fragrans* Wood. (basket plant) is an all-green plant of the Commelinaceae family. Its leaves and runners contain phenolic compounds such as flavonoids and phytosteroids. *C. fragrans* is traditionally used to treat burns, arthritis, skin and oncological diseases, tuberculosis, and asthma [25]. Amongst the phenolic compounds reported to be present in *C. fragrans* are gallic acid, caffeic acid, quercetin, scopoletin, and chicoric acid [26].

Thus far, antioxidant and antibacterial activity studies on plants from the Commelinaceae family have been relatively limited. The antioxidant and antibacterial activity of *R. spathacea* variegata, *R. bermudensis*, *T. pallida*, and *T. zebrina* leaves have never been reported. The antioxidant activity of the juice pressed from *C. fragrans* has been reported by Misin and Sazhina [27] and Olennikov *et al.* [28]. However, the antioxidant and antibacterial activity of *C. fragrans* methanolic leaf extracts has never been reported. This is therefore the first report of the antioxidant and antibacterial activity of the methanolic leaf extracts for all five Commelinaceae plants.

## 2. Materials and Methods

### 2.1. Samples

Fresh leaf samples of all five species were obtained from Selangor, Malaysia, within a 10 km radius. All plants were grown under similar conditions: a monthly mean minimum temperature from 20.8 °C to 25.0 °C, with a monthly mean maximum temperature from 29.6 °C to 32.8 °C; grown on soil; exposed to sunlight (mean daily solar radiation of 19.70 MJ/m^2^) and rain (300–400 mm/month) [29]; and with no fertilizer. For the antioxidant tests, for each species, leaves of similar size were collected from three different individual plants (*n* = 3).

Bacterial isolates were obtained from American Type Culture Collection (ATCC), with the exception of *Proteus vulgaris,* which was obtained from the Institute of Medical Research (IMR), Malaysia. A total of 12 strains of bacteria were used: six Gram-positive bacteria (*Bacillus cereus* (ATCC 14579), *Bacillus subtilis* (ATCC 8188), *Enterococcus faecalis* (ATCC 29212), *Micrococcus luteus* (ATCC 4698), methicillin-resistant *Staphylococcus aureus* (ATCC 33591), and *Staphylococcus epidermidis* (ATCC 12228)) and six Gram-negative bacteria (*Aeromonas hydrophila* (ATCC 49140), *Enterobacter aerogenes* (ATCC 13048), *Pseudomonas aeruginosa* (ATCC 10145), *Proteus mirabilis* (ATCC 12453), *Proteus vulgaris* (clinical), and *Salmonella enterica* Typhimurium (ATCC 14028)). All bacteria were grown on nutrient agar at 37 °C.

### 2.2. Chemicals and Reagents

The various reagents used throughout this project were purchased from suppliers as follows. TPC analysis: Folin-Ciocalteu’s phenol reagent (2N, R and M Chemicals, Essex, UK), gallic acid (98%, Fluka, Steinheim, France), anhydrous sodium carbonate (99%, J. Kollin, UK), diphenyl-2-picrylhydrazyl (DPPH) assay: 2,2-diphenyl-1-picrylhydrazyl (90%, Sigma, St. Louis, MO, USA), ferric reducing power (FRP) assay: ferric chloride hexa-hydrate (100%, Fisher Scientific, Loughborough, UK), potassium ferricyanide (99%, Unilab, Auburn, Australia), trichloroacetic acid (99.8%, HmbG Chemicals, Barcelona, Spain), potassium dihydrogen orthophosphate (99.5%, Fisher Scientific), dipotassium hydrogen phosphate (99%, Merck, Darmstadt, Germany), iron chloride (99%, RandM Chemicals), ferrous ion chelating (FIC) assay: ferrozine (98%, Acros Organics, Morris Plains, NJ, USA), ferrous sulphate hepta-hydrate (HmbG Chemicals), ethylenediaminetetraacetic (99.5%, Bendosen Laboratory Chemicals, Bendosen, Norway), potassium acetate (99%, R and M chemicals), rutin (98%, Sigma), phytochemical screening: sulfuric acid (95%–97%, HmBG Chemicals), hydrochloric acid (37%, Merck, Darmstadt, Germany), Dragendorff reagent (Fluka), α-naphthol (99%, Sigma), antimicrobial activity: nutrient broth (Oxoid, Hampshire, England), nutrient agar (Oxoid, Hampshire, England), and vancomycin (Sigma).

### 2.3. Extraction of Samples

The fresh leaves were gently washed and dabbed dry before being processed. One gram of leaf sample was subjected to liquid nitrogen-aided crushing with a mortar and pestle, followed by extraction with 50 mL of solvent for an hour at room temperature. Different compositions of methanol (50%, 70%, or 100%) were chosen for the extraction from the different species based on the best extraction efficiency (as determined by TPC). *R. bermudensis* and *C. fragrans* were extracted with 50% methanol (84.2% and 88.6% extraction efficiency, respectively), *R. spathacea* variegata with 70% methanol (89.9% extraction efficiency), and *T. zebrina* and *T. pallida* with 100% methanol (92.3% and 83.1% extraction efficiency, respectively). Extracts were filtered and stored at 8 °C when not in use. All analyses were conducted in triplicates.

### 2.4. Determination of Total Phenolic Content (TPC)

The TPC assay was modified from [30] utilizing the Folin–Ciocalteu reagent. Samples (300 μL, in triplicate) were mixed with 1.5 mL of the 10% Folin–Ciocalteu reagent, followed by an addition of 1.2 mL of 7.5% (w/v) sodium carbonate (Na_2_CO_3_) solution. The test tubes were then left to stand for 30 min in the dark at room temperature before the absorbance values were measured at 765 nm with a Hitachi U-1800 spectrophotometer (Shimadzu Corporation, Kyoto, Japan). The total phenolic content was expressed as mg gallic acid equivalent per 100 g of fresh sample (mg GAE/100 g).

### 2.5. DPPH Free Radical Scavenging (FRS) Assay

The DPPH assay was based on the procedures described in Leong and Shui [31] and Miliauskas *et al.* [32], where the reduction of the DPPH (2,2-diphenyl-1-picrylhydrazyl) radical was measured spectrophotometrically to determine the radical scavenging activity of the extract. Two mL of DPPH solution (5.9 mg in 100 mL methanol) was added to 1 mL of three different concentrations of the sample extract. The absorbance of the solution was measured at 517 nm after a 30 min incubation time. The free radical scavenging (FRS) activity was expressed as ascorbic acid (AA) equivalent antioxidant capacity (mg AA/100 g) using the equation: FRS = IC_50(AA)_/IC_50(sample)_ × 10^5^. IC_50(AA)_ was 0.00387 mg/mL [33].

### 2.6. Ferric Reducing Power (FRP) Assay

The FRP was determined with potassium ferricyanide, as in the procedure described by Tan and Chan [34]. This assay assessed the ability of any antioxidants present in the extracts to reduce ferric ions (Fe^3+^) to ferrous ions (Fe^2+^). One mL of sample extract of different dilutions was added with 2.5 mL of 0.2 M phosphate buffer (pH 6.6) and 2.5 mL of 1% (w/v) potassium ferricyanide. The mixture was incubated in a 50 °C water bath for 20 min. Subsequently, 2.5 mL of 10% (w/v) trichloroacetic acid was added to stop the reaction. Next, the mixture in each test tube was separated into aliquots of 2.5 mL, added to 2.5 mL of miliQ water and 0.5 mL of 0.1% (w/v) FeCl_3_. The mixtures were incubated at room temperature for 30 min before the absorbance was measured at 700 nm. FRP was expressed as mg gallic acid equivalent per gram of sample, mg GAE/g using a gallic acid standard curve.

### 2.7. Ferrous Ion Chelating (FIC) Assay

The ferrous ion chelating activity of the extract was based on the procedures described in Mau *et al.* [35], and Singh and Rajini [36]. One mL of 0.1 mM FeSO_4_ was added to 1 mL of sample of different dilutions (0.2, 0.5, and 1 mL of extract, corresponding to 4 mg, 10 mg, and 20 mg of leaf material, respectively), followed by 1 mL of 0.25 mM ferrozine. The mixtures were incubated at room temperature for 10 min before the absorbance was measured at 562 nm. It was expressed as the percentage of iron chelating activity. EDTA (0.017–0.067 mg/mL) was used as a positive control.

### 2.8. Determination of Total Flavonoid Content (TFC)

The flavonoid content was determined with the aluminum chloride colorimetric method as described in Chew *et al.* [37]. Equal volumes of 10% aluminum chloride and 1.0 M potassium acetate (0.1 mL each) were added to 0.5 mL of extract, followed by 2.8 mL of distilled water. The solutions were mixed well and incubated at room temperature for 30 min before the absorbance was taken at 435 nm. The flavonoid concentration was expressed as mg rutin equivalent per 100 g sample, mg RE/100 g.

### 2.9. Total Tannin Content (TTC)

Total tannins were determined based on the method described by Makkar *et al.* [38], which compares the TPC extracts treated with polyvinylpolypyrrolidone (PVPP) with those untreated. Tannins, being protein-binding phenolic compounds, would bind to the PVPP.

The TPC was determined via the Folin–Ciocalteau method as detailed previously. For PVPP treatment, 1 mL of distilled water was added to 100 mg PVPP before adding 1 mL of extract. The mixture was vortexed, incubated at 4 °C for 15 min, vortexed again, and centrifuged at 3000 g for 10 min. The supernatant (consisting of simple phenolics other than tannins) was collected and the phenolic content was determined using the TPC assay. Tannic acid was used to plot the standard curve. Results were expressed as mg equivalent per 100 g sample (mg TAE/100 g).

### 2.10. Determination of Antimicrobial Activity

The minimum inhibitory concentration (MIC) of the samples was determined using the broth microdilution technique in 96-well flat bottom microtiter plates as described by the Clinical and Laboratory Standards Institute [39], with a few modifications. Nutrient broth (180 μL) was loaded into all the wells of the first column of the 96-well plate, followed by 100 μL of nutrient broth in all the other wells. Twenty μL of each sample type (200 mg/mL stock concentration of leaf extract) was loaded into the first column in triplicate, enabling two samples to be run concurrently on a single plate. Serial doubling dilution was then performed nine times, keeping the volume of each well at 100 μL. One hundred μL of nutrient broth inoculated with bacteria the day before was standardized with the McFarland standard and then loaded into these wells for a final working concentration of sample ranging from 10 mg/mL to 0.02 mg/mL. The plates were then incubated overnight. The lowest concentration, where complete inhibition was observed with the unaided eye, was noted as the MIC. Vancomycin (10–0.02 mg/mL) was used as a positive control.

### 2.11. Statistical Analysis

One-way analysis of variance (ANOVA) and a post-hoc Tukey range test were performed to determine significance. A probability value of *p* < 0.05 was considered significant. Analysis was done using SPSS 16 (SPSS Inc., Chicago, IL, USA).

## 3. Results and Discussion

According to Table 1, *T. zebrina* exhibited the highest TPC, TTC, TFC, FRS, and FRP of all the plants tested in this study. *T. zebrina* methanolic leaf extracts also contained higher TPC, FRS, FRP, and TFC than *R. spathacea* (Swartz) Stearn methanolic leaf extracts [10], showing the highest antioxidant content amongst the Commelinaceae reported to date. Moreover, *T. zebrina* has a TPC comparable to lemon myrtle tea, which was previously reported as the herbal tea with the highest TPC in a study comparing 18 tropical and temperate herbal teas [33]; our extract was prepared from 1 g fresh leaf while the lemon myrtle decoction was prepared from 1 g powder. This finding is relevant, as *T. zebrina* is traditionally used to treat gastrointestinal cancer and disorders [8], and consumed as a tea called “Matali” in Mexico. It is therefore worthy of further investigation and promotion as an herbal tea. In addition, approximately 82% of its phenolic content consisted of tannins, a class of phenolic compounds with potent antioxidant and antibacterial activities [40].

**Table 1:** Total phenolic content (TPC), total tannin content (TTC), total flavonoid content (TFC), free radical scavenging (FRS), and ferric reducing power (FRP) of the various Commelinaceae leaf extracts.

Species	TPC (mg GAE/100 g) *	TTC (mg TAE/100 g) *	TFC (mg RE/100 g)	FRS (mg AA/100 g)	FRP (mg GAE/g)
*R. spathacea* variegata	203.9 ± 16.3 ^a,d^ (24.8 ± 2.2) ^a^	20.6 ± 2.3 ^a^	10.8 ± 2.9 ^a^	177.3 ± 15.3 ^a^	1.4 ± 0.1 ^a^
*R. bermudensis*	296.6 ± 21.7 ^b^ (33.5 ± 2.4) ^b^	11.5 ± 1.7 ^b^	18.4 ± 2.4 ^b^	462.2 ± 97.3 ^b^	2.7 ± 0.3 ^b^
*T. zebrina*	620.9 ± 39.7 ^c^ (70.1 ± 4.5) ^c^	57.6 ± 3.5 ^c^	17.1 ± 2.8 ^b^	906.5 ± 88.2 ^c^	4.8 ± 0.3 ^c^
*T. pallida*	153.1 ± 21.8 ^d^ (17.8 ± 2.3) ^d^	13.6 ± 2.1 ^b^	10.6 ± 4.0 ^a^	103.0 ± 36.9 ^a^	0.9 ± 0.1 ^a^
*C. fragrans*	269.3 ± 33.8 ^a,b^ (29.3 ± 2.9) ^a,b^	19.7 ± 1.5 ^a^	2.5 ± 1.4 ^a^	262.5 ± 67.2 ^a^	1.5 ± 0.3 ^a^

Results are expressed as mean ± S.D. (*n* = 3); For each column, values followed by the same letter are not significantly different at *p* < 0.05 as measured by the Tukey HSD test; All gram measurements are expressed in terms of fresh weight; * Values in parentheses are in terms of mg TAE/100 g.

*C. fragrans* and *R. bermudensis* had the second-highest TPC, which was half that of *T. zebrina*. The antioxidant content of the juice pressed from *C. fragrans* leaves has been previously reported as being 73.2 mg/L of juice sample [27] with an IC_50_ of 1.07 mg/mL, based on the DPPH radical scavenging assay [28]. In comparison, the methanolic leaf extract of *C. fragrans* in our study had an IC_50_ of 1.5 ± 0.4; a comparable result with the previously reported value. On the other hand, comparing the antioxidant content was less straightforward because the juice yield was not reported by Misin and Sazhina [27], and it was unclear if the values reported had factored in the 100-fold dilution of their sample. However, using the juice yield reported by Olennikov *et al.* [28] (0.8 L juice per kilogram of leaves), and assuming that the value reported by Misin and Sazhina [27] was based on the undiluted juice, the antioxidant content of the juice would have been 5.85 mg/100 g. Although this value took into account all antioxidants, including non-phenolic antioxidants, it was over 45-fold lower than our methanolic leaf extract. The TPC of the juice has also been reported as 7.12 μg GAE/mL [26], which is equivalent to 0.6 mg GAE/100 g—almost 450 times lower than the TPC of the methanolic leaf extract. Methanolic extraction of *C. fragrans* is therefore more efficient than squeezing tissues for juice, and the leaves of *C. fragrans* contain a higher phenolic content than the runners reported by Olennikov *et al.* [26].

**Figure 1:**
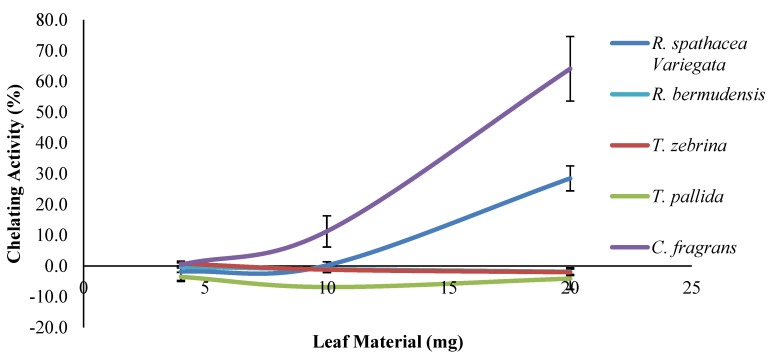
Ferrous ion chelating (FIC) activity of the various Commelinaceae leaf extracts.

The methanolic extract of *C. fragrans* also exhibited by far the highest FIC activity (Figure 1), with a chelating EC_50_ of 17.3 mg leaf material. *R. spathacea* variegata exhibited the second highest FIC, but did not achieve EC_50_ at the concentrations tested, thus indicating reasonably weak FIC activity. The other species exhibited practically no FIC activity. This indicates that the antioxidants present in some of the Commelinaceae leaves lack the ability to chelate ferrous ions when compared to ferrozine and thus have low or no secondary antioxidant activity, which implies the low ability of the extracts to prevent the Fenton reaction. This shows that the primary antioxidant activity of a plant (as measured by the FRS and FRP assays) does not necessarily reflect its secondary antioxidant activity due to the differences in mechanisms [41]. The primary antioxidant activities of the five species did, however, show a strong positive correlation with the TPC (FRS: *R* = 0.98, *p* < 0.001; FRP: *R* = 0.97, *p* < 0.001) and a good positive correlation with the TTC (FRS: *R* = 0.85, *p* < 0.001; FRP: *R* = 0.85, *p* < 0.001), but not with the TFC (FRS: *R* = 0.61, *p* = 0.020; FRP: *R* = 0.65, *p* = 0.012). This shows that tannins play a stronger role than flavonoids in single electron transfer-based primary antioxidant activity.

*R. bermudensis* and *R. spathacea* variegata showed: (1) lower TPC, FRS, and FRP and (2) higher TFC than *R. spathacea* (Swartz) Stearn [10], despite being closely related. Almost 80% of *R. spathacea* variegata’s phenolic compounds are in the form of tannins, while only a third of the phenolic compounds in *R. bermudensis* were tannins. Although the TPC of *R. bermudensis* was similar to *C. fragrans* and not much higher than *R. spathacea* variegata, *R. bermudensis* exhibited exceptionally high FRS and FRP activity. It is therefore likely that the phenolic compounds present in *R. bermudensis* are more effective antioxidants and free radical scavengers than those present in the other species.

All five Commelinaceae species demonstrated antibacterial activity against most of the strains, although the six Gram-negative strains proved more resistant to treatment, with only two showing susceptibility at an extract concentration of 10 mg/mL or below (Table 2). The lower susceptibility of the Gram-negative strains can be attributed to their higher outer layer impermeability [42], an observation consistent with the antibacterial activity of *R. spathacea* (Swartz) Stearn [10]. Of the five Commelinaceae species, *T. zebrina* exhibited the best antibacterial activity against Gram-positive bacteria, with a MIC of 5 mg/mL against three strains, while the other four Commelinaceae leaf extracts had a MIC of 5 mg/mL on fewer than three strains. *T. zebrina* also showed antibacterial activity against two of the six Gram-negative strains, with a MIC of 5 mg/mL. The superior antibacterial activity of *T. zebrina* compared to the other tested species may be attributed to its exceptionally high phenolic and tannin content. The superior antioxidant and antibacterial activity of *T. zebrina* compared to the other Commelinaceae may explain several of its ethnopharmalogical applications, particularly its anti-inflammatory and anti-infective activity [43,44]. Surprisingly, *T. pallida* was similarly active against Gram-negative bacteria (MIC of 5 mg/mL against the same two Gram-negative strains) despite having the lowest antioxidant content and activity. It is likely that the phenolic compounds present may be more potent anti-Gram-negative agents in *T. zebrina* than in *T. pallida*, and/or the anti-Gram-negative compounds present may not necessarily be phenolics (for example, terpenoids or alkaloids). The leaves of *T. zebrina* and *T. pallida* warrant future isolation and identification work given their activity against Gram-negative bacteria, as the isolated compound(s) are likely to have a lower MIC than the crude extracts used in this experiment.

**Table 2:** MIC of Commelinaceae leaf extracts against 12 species of bacteria.

Bacterium	*R. spathacea* Variegata	*R. bermudensis*	*T. zebrina*	*T. pallida*	*C. fragrans*
Gram-Positive					
*Bacillus cereus* (ATCC 14579)	>10	10	5	5	5
*Bacillus subtilis* (ATCC 8188)	>10	10	10	10	5
*Micrococcus luteus* (ATCC 4698)	>10	10	5	10	10
Methicillin-Resistant *Staphylococcus aureus* (ATCC 33591)	>10	10	5	10	10
*Staphylococcus epidermidis* (ATCC 12228)	5	10	>10	>10	>10
*Enterococcus faecalis* (ATCC 29212)	10	>10	>10	5	>10
Gram-Negative					
*Aeromonas hydrophila* (ATCC 49140)	5	>10	5	5	5
*Proteus vulgaris* (Clinical)	>10	>10	5	5	>10

MIC expressed as mg/mL; Vancomycin MIC < 0.02 mg/mL; *P. aeruginosa* (ATCC 10145), *P. mirabilis* (ATCC 12453), *S. typhimurium* (ATCC 14028), and *E. aerogenes* (ATCC 13048) have MIC exceeding 10 mg/mL for all the crude fractions.

## 4. Conclusions

*T. zebrina* exhibited the highest antioxidant content and antioxidant activity of the five Commelinaceae plants studied, higher than the previously-reported antioxidant content and activity of *R. spathacea* (Swartz) Stearn leaves. All five leaf extracts exhibited varying degrees of antibacterial activity against many of the bacteria tested, with *T. zebrina* being the most active against Gram-positive and Gram-negative bacteria.

## References

[1] Edeoga H.O., Ogbebor N.O. (1999). Distribution of calcium oxalate crystals in some Nigerian species of *Aneilema* R. Br. (Commelinaceae). Plant Biosyst..

[2] Satterfield S.K., Mertens T.R. (1972). *Rhoeo spathacea*: A tool for teaching meiosis and mitosis. J. Hered..

[3] Faden R.B., Kubitzki K. (1998). Commelinaceae. Flowering Plants—Monocotyledons.

[4] Wilson A.K. (1981). Commelinaceae—A review of the distribution, biology and control of the important weeds belonging to this family. Trop. Pest Manag..

[5] González-Avila M., Arriaga-Alba M., de la Garza M., del Carmen HernándezPretelín M., Domínguez-Ortíz M.A., Fattel-Fazenda S., Villa-Treviño S. (2003). Antigenotoxic, antimutagenic and ROS scavenging activities of a *Rhoeo discolor* ethanolic crude extract. Toxicol. In Vitro.

[6] Arriaga-Alba M., Blasco J.L., Ruíz-Pérez N.J., Sánchez-Navarrete J., Rivera-Sánchez R., González-Avila M. (2011). Antimutagenicity mechanisms of the *Rhoeo discolor* ethanolic extract. Exp. Toxicol. Pathol..

[7] Mensah A.Y., Houghton P.J., Dickson R.A., Fleischer T.C., Heinrich M., Bremner P. (2006). *In vitro* evaluation of effects of two Ghanaian plants relevant to wound healing. Phytother. Res..

[8] Alonso-Castro A.J., Villarreal M.L., Salazar-Olivo L.A., Gomez-Sanchez M., Dominguez F., Garcia-Carranca A. (2011). Mexican medicinal plants used for cancer treatment: Pharmacological, phytochemical and ethnobotanical studies. J. Ethnopharmacol..

[9] Perry C.C., Müller W.E.G., Grachev M.A. (2009). An overview of silica in biology: Its chemistry and recent technological advances. Biosilica in Evolution, Morphogenesis, and Nanobiotechnology.

[10] Tan J.B.L., Lim Y.Y., Lee S.M. (2013). Antioxidant and antibacterial activity of *Rhoeo spathacea* (Swartz) Stearn leaves. J. Food Sci. Technol..

[11] Xeriscape Plants. http://www.ctahr.hawaii.edu/oc/freepubs/pdf/OF-42.pdf.

[12] Golczyk H. (2011). Cytogenetics of the permanent translocation heterozygote *Rhoeo spathacea* var. *variegata*. Implications for complex chromosome rearrangements in *Rhoeo*. Caryologia.

[13] Paiva E.A.S., Isaias R.M.S., Vale F.H.A., Queiroz C.G.S. (2003). The influence of light intensity on anatomical structure and pigment contents of *Tradescantia pallida* (Rose) Hunt. cv. *purpurea* Boom (Commelinaceae) leaves. Braz. Arch. Biol. Technol..

[14] Rainho C.R.D., Kaezer A., Aiub C.A.F., Felzenszwalb I. (2010). Ability of *Allium cepa* L. root tips and *Tradescantia pallida* var. *purpurea* in *N*-nitrosodiethylamine genotoxicity and mutagenicity evaluation. An. Acad. Bras. Ciênc..

[15] Misik M., Ma T.H., Nersesyan A., Monarca S., Kim J.K., Knasmueller S. (2011). Micronucleus assays with *Tradescantia* pollen tetrads: An update. Mutagenesis.

[16] Thewes M.R., Junior D.E., Droste A. (2011). Genotoxicity biomonitoring of sewage in two municipal wastewater treatment plants using the *Tradescantia pallida* var. *purpurea* bioassay. Genet. Mol. Biol..

[17] Yang D.S., Pennisi S.V., Son K.C., Kays S.J. (2009). Screening indoor plants for volatile organic pollutant removal efficiency. HortScience.

[18] Li T.S.C. (2006). Taiwanese Native Medicinal Plant: Phytopharmacology and Therapeutic Values.

[19] Faden R.B. (2008). The author and typification of *Tradescantia zebrina* (Commelinaceae). Kew Bull..

[20] Yanzhi M. (2009). Pigment content and anatomical structure of leaves of several species of red-leafed plants. J. N. E. Forest. Univ..

[21] Glimn-Lacy J., Kaufman P.B. (2006). Spiderwort family (Commelinaceae). Botany Illustrated.

[22] Amaral F.M.M., Ribeiro M.N.S., Barbosa-Filho J.M., Reis A.S., Nascimento F.R.F., Macedo R.O. (2006). Plants and chemical constituents with giardicidal activity. Braz. J. Pharmacog..

[23] González-Coloma A., Reina M., Sáenz C., Lacret R., Ruiz-Mesia L., Arán V.J., Sanz J., Martinez-Diaz R.A. (2012). Antileishmanial, antitrypanosomal, and cytotoxic screening of ethnopharmacologically selected Peruvian plants. Parasitol. Res..

[24] Alaba C.S.M., Chichioco-Hernandez C.L. (2014). 15-Lipoxygenase inhibition of Commelina benghalensis, Tradescantia fluminensis, Tradescantia zebrina. Asian Pac. J. Trop. Biomed..

[25] Chernenko T.V., Ul’chenko N.T., Glushenkova A.I., Redzhepov D. (2007). Chemical investigation of *Callisia fragrans*. Chem. Nat. Compd..

[26] Olennikov D.N., Ibragimov T.A., Zilfikarov I.N., Chelombit’ko V.A. (2008). Chemical composition of *Callisia fragrans* juice 1. Phenolic compounds. Chem. Nat. Compd..

[27] Misin V.M., Sazhina N.N. (2010). Content and activity of low-molecular antioxidants in juices of medicinal plants. Russ. J. Phys. Chem. B.

[28] Olennikov D.N., Zilfikarov I.N., Toropova A.A., Ibragimov T.A. (2008). Chemical composition of *Callisia fragrans* wood. juice and its antioxidative activity (*in vitro*). Chem. Plant Raw Mater..

[29] Malaysian Meteorological Department. http://www.met.gov.my/index.php?option=com_content&task=view&id=34&Itemid=1586.

[30] Kähkönen M.P., Hopia A.I., Vuorela H.J., Rauha J.P., Pihlaja K., Kujala T.S., Heinonen M. (1999). Antioxidant activity of plant extracts containing phenolic compounds. J. Agric. Food Chem..

[31] Leong L.P., Shui G. (2002). An investigation of antioxidant capacity of fruits in Singapore markets. Food Chem..

[32] Miliauskas G., Venskutonis P.R., van Beek T.A. (2004). Screening of radical scavenging activity of some medicinal and aromatic plant extracts. Food Chem..

[33] Chan E.W.C., Lim Y.Y., Chong K.L., Tan J.B.L., Wong S.K. (2010). Antioxidant properties of tropical and temperate herbal teas. J. Food Comp. Anal..

[34] Tan Y.P., Chan E.W.C. (2014). Antioxidant, antityrosinase and antibacterial properties of fresh and processed leaves of *Anacardium occidentale* and *Piper betle*. Food Biosci..

[35] Mau J.L., Lai E.Y.C., Wang N.P., Chen C.C., Chang C.H., Chyau C.C. (2003). Composition and antioxidant activity of the essential oil from *Curcuma zedoaria*. Food Chem..

[36] Singh N., Rajini P.S. (2004). Free radical scavenging activity of an aqueous extract of potato peel. Food Chem..

[37] Chew Y.L., Goh J.K., Lim Y.Y. (2009). Assessment of *in vitro* antioxidant capacity and polyphenolic composition of selected medicinal herbs from Leguminosae family in peninsular Malaysia. Food Chem..

[38] Makkar H.P.S., Siddhuraju P., Becker K. (2007). Plant Secondary Metabolites.

[39] Clinical and Laboratory Standards Institute (2009). Methods for dilution antimicrobial susceptibility tests for bacteria that grow aerobically; approved standard—Eight edition. CLSI Doc..

[40] Michel T., Destandau E., Le Floch G., Lucchesi M.E., Elfakir C. (2012). Antimicrobial, antioxidant and phytochemical investigations of sea buckthorn (*Hippophaë rhamnoides* L.) leaf, stem, root and seed. Food Chem..

[41] Lai H.Y., Lim Y.Y. (2011). Evaluation of antioxidant activities of the methanolic extracts of selected ferns in Malaysia. Int. J. Environ. Sci. Dev..

[42] Russell A.D. (1999). Bacterial resistance to disinfectants: Present knowledge and future problems. J. Hosp. Infect..

[43] Frei B., Heinrich M., Bork P.M., Herrmann D., Jaki B., Kato T., Kuhnt M., Schmitt J., Schühly W., Volken C. (1998). Multiple screening of medicinal plants from Oaxaca, Mexico: Ethnobotany and bioassays as a basis for phytochemical investigation. Phytomedicine.

[44] Tene V., Malagón O., Finzi P.V., Vidari G., Armijos C., Zaragoza T. (2007). An ethnobotanical survey of medicinal plants used in Loja and Zamora-Chinchipe, Ecuador. J. Ethnopharmacol..

